# Supplementary material for: Asynchronous magnetic resonance elastography: Shear wave speed reconstruction using noise correlation of incoherent waves
Source: Magn Reson Med. 2022 Oct 27;89(3):990–1001. doi: 10.1002/mrm.29502 (PMC9792433; doi:10.1002/mrm.29502)
Supplement: Supplementary file 1 — DATA S1 MATLAB code used to generate the numerical simulations of Figure 1 [file MRM-89-990-s002.zip › k-Wave/helpfiles/minND.html]

minND :: Functions (k-Wave)


# minND

Return the value and indices of the smallest value in an N-D array.

## Syntax

```
min_val = minND(mat)
[min_val, ind] = minND(mat)
```

## Description

`minND` returns the smallest value in the N-D array given by `mat`. The indices of the smallest value within the array can also be returned, where `ind = [ind_dim_1, ind_dim_2, ... , ind_dim_N]`. The indices can be used to directly address the smallest value, i.e., `min_val = mat(ind)`.

## Inputs

|  |  |
| --- | --- |
| `mat` | input array |

## Outputs

|  |  |
| --- | --- |
| `min_val` | smallest value in `mat` |
| `ind` | array of indices corresponding to the location of the smallest value in `mat` |
